# Zoledronate promotes ECM degradation and apoptosis via Wnt/β-catenin

**DOI:** 10.1515/med-2022-0463

**Published:** 2022-04-18

**Authors:** Jialing Xiao, Yali Li, Gang Cheng, Guochao Xu

**Affiliations:** Department of Stomatology, Zhejiang Hospital, Hangzhou 310013, Zhejiang, China; Department of Dermatology, The First Affiliated Hospital, Zhejiang University School of Medicine, Hangzhou, Zhejiang, China; Department of Stomatology, Zhejiang Provincial People’s Hospital, People’s Hospital of Hangzhou Medical College, No. 158 Shangtang Road, Hangzhou 310014, Zhejiang, China; Department of Stomatology, Zhejiang Hospital, No. 12 Lingyin Road, Hangzhou 310013, Zhejiang, China

**Keywords:** temporomandibular joint, osteoarthritis, zoledronate, extracellular matrix, apoptosis, Wnt signaling pathway

## Abstract

This study examined the potential mechanism of zoledronate on interleukin (IL)-1β-induced temporomandibular joint osteoarthritis (TMJOA) chondrocytes, using IL-1β-induced rabbit immortalized mandibular condylar chondrocytes cultured with zoledronate. Cell viability, apoptosis, mRNA, and protein expression of relevant genes involved in extracellular matrix (ECM) degradation, apoptosis, and Wnt/β-catenin signaling were examined. The involvement of the Wnt/β-catenin signaling was examined using Wnt/β-catenin inhibitor (2-(4-(trifluoromethyl)phenyl)-7,8-dihydro-5H-thiopyrano[4,3-d]pyrimidin-4-ol (XAV-939)) and activator lithium chloride (LiCl). Aggrecan and type II collagen were downregulated by zoledronate, especially with 100 nM for 48 h (*p* < 0.01), consistently with the upregulation of A disintegrin and metalloproteinase with thrombospondin motifs-4 (ADAMTS-4) (*p* < 0.001), matrix metalloprotease-9 (MMP-9) (*p* < 0.01), caspase-3 (*p* < 0.001) and downregulation of proliferating cell nuclear antigen (PCNA) (*p* < 0.01). The apoptotic rate increased from 34.1% to 45.7% with 100 nM zoledronate for 48 h (*p* < 0.01). The effects of zoledronate on ADAMTs4 (*p* < 0.001), MMP-9 (*p* < 0.001), caspase-3 (*p* < 0.001), and PCNA (*p* < 0.01) were reversed by XAV-939, while LiCl increased caspase-3 expression (*p* < 0.01). In conclusion, zoledronate enhances IL-1β-induced ECM degradation and cell apoptosis in TMJOA chondrocytes. Wnt/β-catenin signaling might be involved in this process, but additional studies are necessary to determine the exact involvement of Wnt/β-catenin signaling in chondrocytes after zoledronate treatment.

## 1 Introduction

Osteoarthritis (OA) is a degenerative disease characterized by progressive cartilage damage, bone sclerosis, osteophyte formation, and chronic pain [1]. Temporomandibular joint (TMJ) osteoarthritis (TMJOA) is an important subtype of temporomandibular disorders (TMDs). The etiology of TMJOA is complex and multifactorial; it is often idiopathic since the TMJ performs the most complicated movement in the human body [2,3]. Inflammatory cytokines are increased in the synovial fluid of patients with TMJOA or animal models, suggesting the importance of inflammation in the progression of TMJOA [4,5]. The catabolic enzymes in the cartilage matrix that are involved in the degradation of the cartilage and destruction of subchondral bone are upregulated during the progression of TMJOA [6].

Interleukin (IL)-1β plays a pivotal role in cartilage destruction during the pathophysiological process of OA by promoting the release of degenerative matrix enzymes and inhibiting the synthesis of the extracellular matrix (ECM) proteins by chondrocytes [7]. IL-1β can induce the secretion of matrix metalloproteases (MMPs) by chondrocytes, leading to ECM degradation and increased chondrocyte apoptosis [8,9]. Members of the A disintegrin and metalloproteinase with thrombospondin motifs (ADAMTS) family are more efficient than MMPs to cleave the aggrecan core protein [11]. Aggrecan and type II collagen are important components of the ECM in TMJ condyle cartilage [10,11]. Therefore, IL-1β is usually used to induce chondrocyte degradation and apoptosis to study OA. Several lines of evidence derived from animal models support the involvement of the Wnt/β-catenin signaling pathway in the molecular mechanisms underlying cartilage degradation [12–15], as well as a potential treatment target against OA [16].

Bisphosphonates (BPs) are nonhydrolyzable analogs of inorganic pyrophosphate and are widely used for the treatment of disorders related to calcium metabolism, including osteoporosis, Paget’s disease, multiple myeloma, and bone metastases [17]. They inhibit bone resorption by inducing osteoclast apoptosis. Nitrogen-containing BPs (pamidronate and risedronate) can protect the chondrocytes from dexamethasone-induced growth retardation and apoptosis [18]. Zoledronate (ZOL) can decrease the expression of vascular endothelial growth factor (VEGF) A in synovial cells, suppressing angiogenesis, reducing inflammatory changes, and alleviating pain [19]. Intra-articular injected ZOL could suppress synovial inflammation but not reduce cartilage degeneration in early OA models [20]. High-dose ZOL by intravenous injection may be chondroprotective in OA animal models [21,22]. On the other hand, BPs neither provide symptomatic relief nor slow down radiographic progression in knee OA [23]. ZOL has antiproliferative and proapoptotic effects in dental pulp stem cells and colorectal cancer cells [24,25]. Nonetheless, few studies examined the effects of ZOL on chondrocytes (especially on TMJOA chondrocytes), ECM degradation, apoptosis, and the mechanisms involved.

Several cases of TMJ disorders such as joint dislocation, ankylosis, destruction, and suppurative arthritis were reported after BP therapy, and BP-related osteonecrosis of the jaw attracted researchers’ attention to the effect of ZOL on maxillofacial bone and cartilage diseases [26–28]. To the best of our knowledge, there is no evidence about the relationship between TMJOA and BP. TMJOA is a kind of OA characterized by the loss of articular cartilage and degradation of the cartilage matrix [1,2]. ZOL is widely used to treat bone-related disorders and could promote growth inhibition and apoptosis [19–22,24,25,27].

Therefore, this study aimed to examine the role and potential mechanisms of ZOL on IL-1β-induced TMJOA chondrocytes. We examined the expression levels of several pivotal genes and corresponding proteins involved in ECM degradation, cell apoptosis, and the Wnt/β-catenin signaling pathway.

## 2 Materials and methods

### 2.1 Cell culture

The rabbit immortalized mandibular condylar chondrocyte (IMCC) cell line used in this study was purchased from the Department of Oral Biology of the School of Stomatology of the Fourth Military Medical University (China). The IMCCs were cultured in high-glucose Dulbecco’s modified Eagle medium (DMEM) (GIBCO, Invitrogen Inc., Carlsbad, CA, USA) supplemented with 10% FBS (Hyclone, Thermo Fisher Scientific, Waltham, MA, USA) and penicillin/streptomycin (GIBCO, Invitrogen Inc., Carlsbad, CA, USA) at 37°C in a 5% CO_2_ atmosphere.

A total of 1 × 10^5^ cell/mL IMCCs were plated on 6-well plates and treated with 10 ng/mL IL-1β (Novoprotein, Shanghai, China) to induce chondrocyte degradation. Samples were collected at different time points (1, 2, 3, 6, 12, and 24 h), and the expression levels of ECM-related genes were analyzed by reverse transcription-quantitative polymerase chain reaction (qRT-PCR), including aggrecan, type II collagen, ADAMTS-4, ADAMTS-5, and MMP-9.

### 2.2 Cell treatments

The chondrocytes were examined under a Leica DM4000B microscope (Leica, IL, USA) for any cellular morphological changes in the presence of the control (DMEM), 10 ng/mL IL-1β alone, or in combination with different concentrations (1 nM, 10 nM, 100 nM, and 1 µM) and times (0, 24, 48, and 96 h) of ZOL (Aladin, Shanghai, China). IL-1β-induced IMCCs were treated with 10 or 100 nM ZOL for 24 or 48 h. Caspase-9 (apoptosis initiator) and caspase-3 (apoptosis effector) were examined by qRT-PCR and western blotting. The percentage of apoptotic cells was determined by flow cytometry. The expression of the proliferating cell nuclear antigen (PCNA) was determined [29]. XAV-939 (Selleck Chemicals, TX, USA) and LiCl (Sigma-Aldrich Co., MO, USA) were used as the inhibitor and activator of the Wnt/β-catenin signaling pathway, respectively, and were added to the ZOL-treated TMJOA chondrocytes for 24 and 48 h. Chondrocytes without ZOL were used as controls. The changes in chondrocyte morphology were examined using an inverted phase microscope (Nikon, Tokyo, Japan).

### 2.3 qRT-PCR

The total RNA was extracted from chondrocytes using Trizol (Invitrogen, Carlsbad, CA, USA), according to the manufacturer’s protocol. The first-strand cDNA was synthesized using the RevertAid First Strand cDNA Synthesis Kit (Thermo Fisher Scientific, Waltham, MA, USA). qRT-PCR was performed on a Roche LightCycler 480 System (Roche, Basel, CH) with the SYBR Green Master Mix (Starbiolab, Hangzhou, China) and primers (Table 1). glyceraldehyde-3-phosphate dehydrogenase (GAPDH) was used as the reference gene.

**Table 1:** Primer sequences used for qPCR

Gene	Primer sequences
Aggrecan	F: TTGGAGGTCGTGGTGAAAGG
	R: TCCCGGATGCCGTAGGTT
Type II Collagen	F: ACGACATAATCTGTGAAGACACCAAGG
	R: TGGCAGTGGCGAGGTCAGTAG
MMP-9	F: GTGAAGACGCAGACGGTGGATTC
	R: GGTACTCACACGCCAGAAGAAGC
ADAMTS-4	F: CTGACCACTTCGACACAGCCATC
	R: GTCCATCATCTTCCACGATAGCACAG
ADAMTS-5	F: AGTGTGGAGTATGCGGAGGAGAC
	R: TTCTTCTTCAAGGCTAAGTAGGCAGTG
PCNA	F: TGAAGATAATGCGGACACCTTGGC
	R: TGGCTGAGGTCTCGGCATATACG
Caspase-3	F: GATTCTAAGCCACGGTGATGAAGGAG
	R: CACTGTCTGTCTCGATGCCACTG
Caspase-9	F: AGCCTGACCTGACCGCCAAG
	R: CAGCCGTGAGACAGGATGACAAC
GAPDH	F: ATGGGGAAGGTGAAGGTCG
	R: TAAAAGCAGCCCTGGTGACC

### 2.4 Western blot

The chondrocytes were lysed in ristocetin-induced platelet aggregation buffer (Beyotime Biotechnology, Shanghai, China) containing a protease and phosphatase inhibitor cocktail (ThermoFisher Scientific, Waltham, MA, USA). The protein concentration was determined using a bicinchoninic acid assay kit (Beyotime Biotechnology, Shanghai, China). Equal amounts of proteins were loaded on 10% sodium dodecyl sulfate-polyacrylamide gel and transferred onto polyvinylidene difluoride membranes (Millipore Corp., Billerica, MA, USA). The membranes were blocked with 5% nonfat milk for 1 h and incubated with primary antibody overnight at 4°C. The following primary antibodies were used to detect the proteins: rabbit anti-aggrecan polyclonal antibody (1:500, Proteintech, Wuhan, China), rabbit anti-collagen II polyclonal antibody (1:500, Proteintech, Wuhan, China), rabbit anti-MMP-9 polyclonal antibody (1:500, Proteintech, Wuhan, China), rabbit anti-ADAMTS-4 polyclonal antibody (1:500, RuiyingBio, Suzhou, China), rabbit anti-Caspase-3 polyclonal antibody (1:500, Proteintech, Wuhan, China), and mouse anti-PCNA monoclonal antibody (1:500, RuiyingBio, Suzhou, China). The membranes were incubated with a goat anti-rabbit IgG conjugated to horseradish peroxidase (1:1,000, Beyotime, Shanghai, China) or a goat anti-mouse IgG conjugated to horseradish peroxidase (1:1,000, Beyotime, Shanghai, China) for 1 h. Incubation with a mouse anti-GAPDH monoclonal antibody (1:1,000, Abcam, Cambridge, UK) was performed as the loading sample control. The blots were revealed using the Clarity MAX™ Western enterochromaffin-like Substrate (BioRad, CA, USA) by ChemiDoc™ XRS + System (BioRad, CA, USA).

### 2.5 Flow cytometry

The chondrocytes were harvested and washed three times with phosphate-buffered saline (PBS) and resuspended in loading buffer (Sangon Biotech, Shanghai, China) to 2–5 × 10^5^ cells/mL. For the apoptosis analysis, the Annexin V-fluorescein isothiocyanate/propidium iodide kit (Sangon Biotech, Shanghai, China) was used strictly according to the manufacturer’s protocol. The Becton, Dickinson (BD) FACSCanto™ II system (BD Biosciences, CO, USA) was used to detect the surface markers of the chondrocytes. The results were analyzed using the FlowJo software.

### 2.6 3-(4,5-Dimethylthiazol-2-yl)-2,5-diphenyltetrazolium bromide (MTT) assay

The MTT viability assay was performed to determine the appropriate concentrations of XAV-939 and LiCl. Chondrocyte viability was investigated using MTT (Sigma St Louis, MO, USA). Briefly, the chondrocytes (5,000 cells/well, 100 µL medium) were cultured in a 96-well plate (Corning, NY, USA) and 20 µL of MTT solution (5 mg/mL in PBS) was added and incubated for 4 h at 37°C. The medium was removed, 100 µL of dimethyl sulfoxide was added (DMSO, Sigma St Louis, MO, USA), and the optical density was measured at 490 nm using a plate reader (Bio-Tek, Winooski, VT, USA).

### 2.7 Statistical analysis

All data were presented as mean ± SEM from three independent experiments and were analyzed using analysis of variance and Student’s *t*-test using SPSS 21 (IBM, Armonk, NY, USA). Plots were drawn using GraphPad Prism 6 (GraphPad Software Inc., San Diego, CA, USA). The *p*-values of < 0.05 were considered statistically significant.

## 3 Results

### 3.1 Induction of IMCC degradation by IL-1β treatment for 6 h

**Figure 1:**
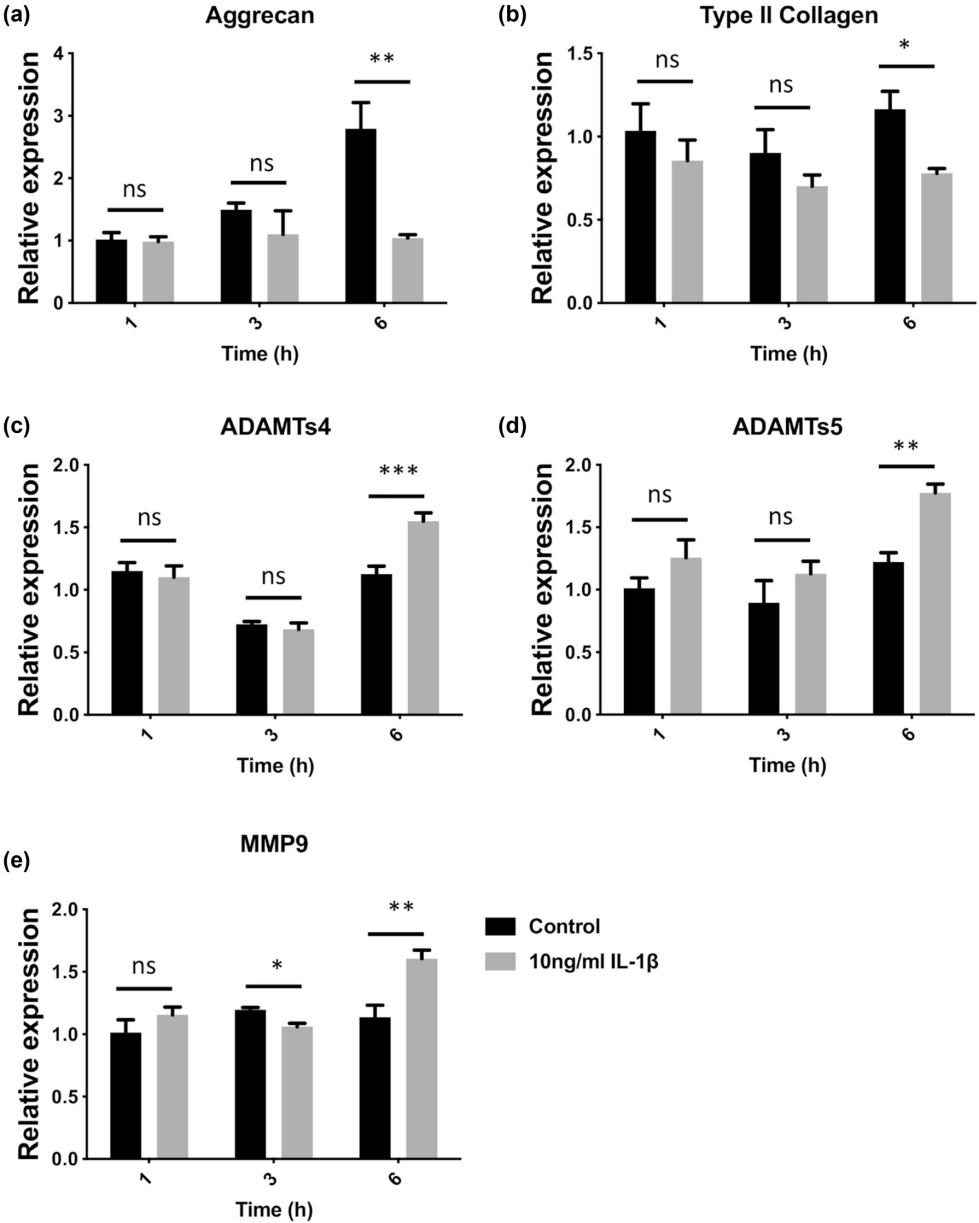
Expression levels of ECM-related genes in IL-1β-treated rabbit IMCCs. Expression levels of (a) aggrecan, (b) type II collagen, (c) ADAMTS-4, (d) ADAMTS-5, and (e) MMP-9 were analyzed by qRT-PCR. All data are presented as mean ± SEM. *p < 0.05, **p < 0.01, ns: not significant.

After rabbit IMCCs were treated with 10 ng/mL of IL-1β, the expression of aggrecan, type II collagen, ADAMTS-4, and ADAMTS-5 did not significantly change after 1 and 3 h of culture (all *p* > 0.05) but were all higher compared with controls at 6 h (all *p* < 0.05) (Figure 1a–d). Compared with controls, the expression of MMP-9 was not significantly different at 1 h, was lower at 3 h (*p* < 0.05), but was higher at 6 h (*p* < 0.01) (Figure 1e). Therefore, the TMJOA cells were treated with 10 ng/mL of IL-1β for 6 h for the subsequent experiments, which could induce a decrease in chondrocytes ECM and upregulation of ADAMTS and MMPs.

### 3.2 Changes in chondrocytes morphology and ECM degradation after ZOL treatment

**Figure 2:**
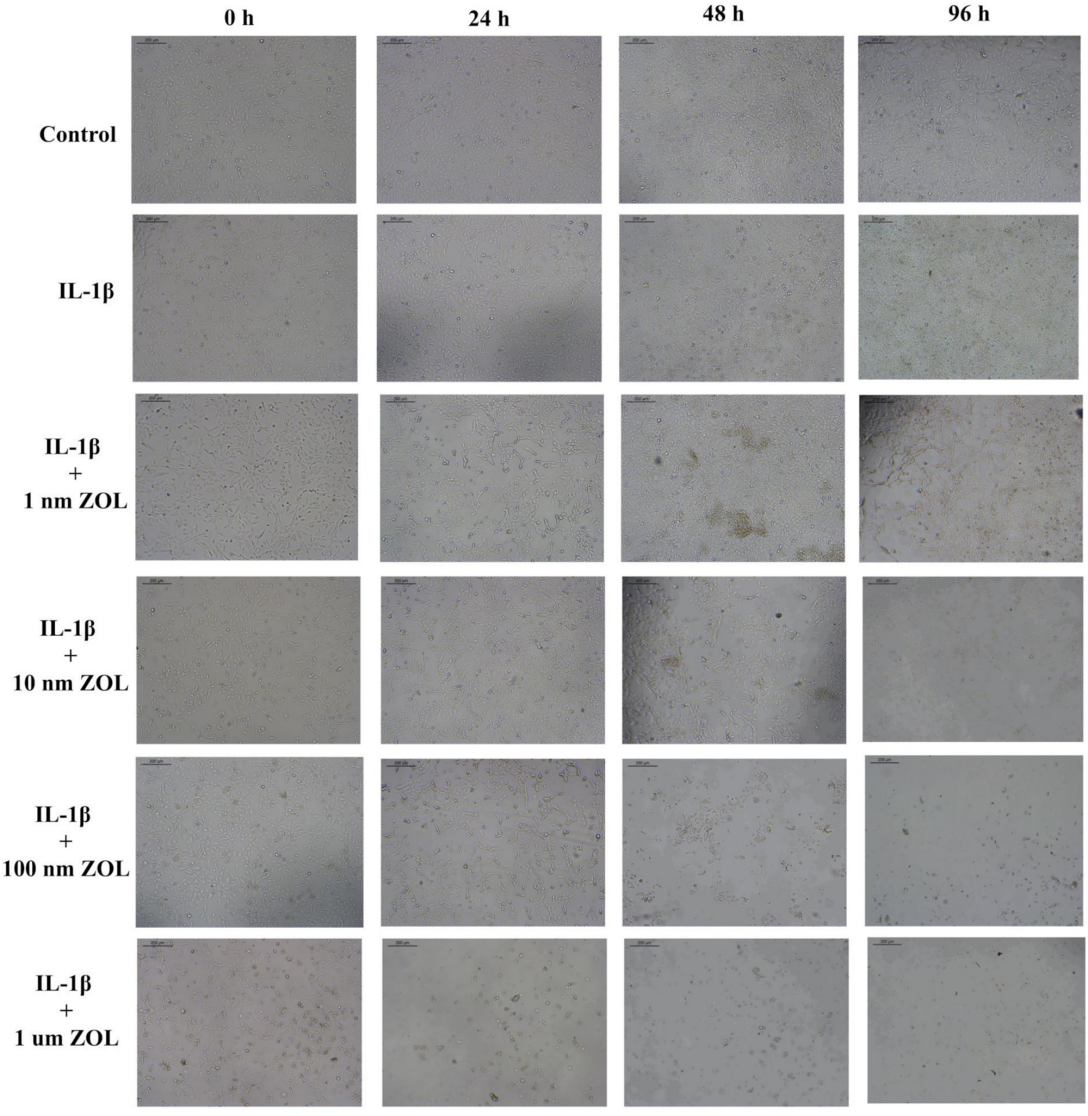
The morphological and abundance changes of TMJOA chondrocytes treated with different concentrations and times of ZOL (scale bar = 200 µm).

**Figure 3:**
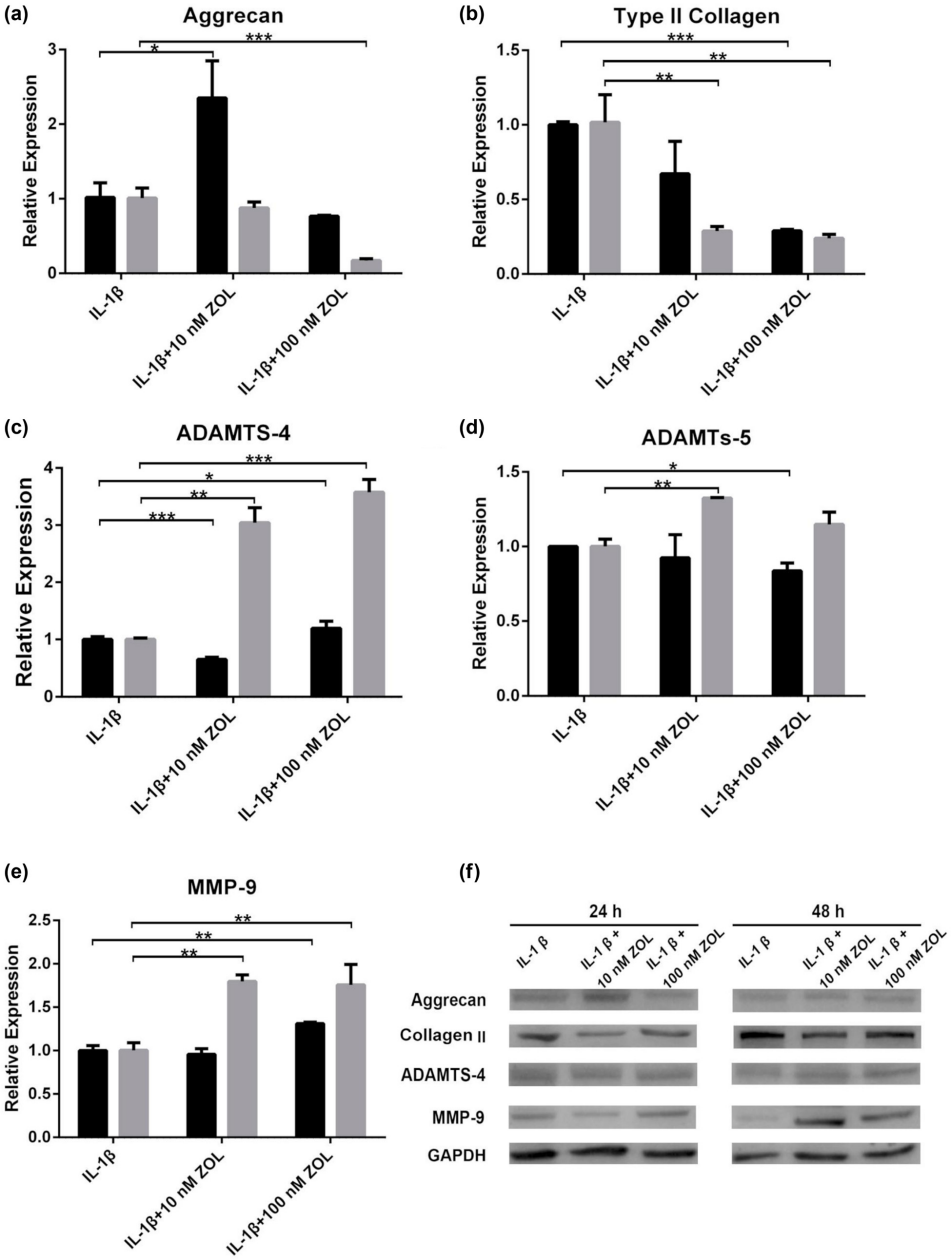
Expression levels of ECM-related genes and proteins after treatment with 10 or 100 nM ZOL in TMJOA chondrocytes. (a) Aggrecan, (b) type II collagen, (c) ADAMTS-4, (d) ADAMTS-5, and (e) MMP-9 were analyzed by qRT-PCR. (f) ECM-related proteins were detected by western blotting. Black bars represent the control group, and gray bars represent the 10 ng/mL IL-1β group. All data are presented as mean ± SEM. *p < 0.05, **p < 0.01, ***p < 0.001 between the two groups linked by the line; all other comparisons are not significant.

The cellular morphology of the chondrocytes was polygonal with clear-cut boundaries and compact arrangement when being untreated or early during 10 ng/mL IL-1β treatment. With the increase of ZOL concentrations and the prolongation of treatment time, the chondrocytes became sparsely arranged, with irregular and elongated shapes. The typical proapoptotic and antiproliferation morphological changes appeared in the presence of 10 or 100 nM ZOL for 24 or 48 h (Figure 2). Figure 3a shows that ZOL at 10 nM for 24 h resulted in an increase in messenger RNA (mRNA) aggrecan expression at 24 h compared with IL-1β controls (*p* < 0.05), while 100 nM ZOL led to a decrease in aggrecan expression at 48 h (*p* < 0.001). Figure 3b shows that ZOL 10 nM led to a decreased mRNA expression of type II collagen at 48 h (*p* < 0.05), while 100 nM ZOL led to a decreased type II collagen expression at 24 (*p* < 0.001) and 48 h (*p* < 0.01). The mRNA expression of ADAMTS-4 first decreased at 24 h with 10 nM ZOL (*p* < 0.001) but increased at 48 h (*p* < 0.01) (Figure 3c), while it was increased with 100 nM ZOL at 24 (*p* < 0.05) and 48 h (*p* < 0.001). The mRNA expression of ADAMTS-5 was increased at 48 h with 10 nM ZOL (*p* < 0.01) and decreased at 24 h with 100 nM ZOL (*p* < 0.05) (Figure 3d). Figure 3e shows that the mRNA expression of MMP-9 was increased with 10 nM ZOL at 48 h (*p* < 0.01), as well as with 100 nM ZOL at 24 (*p* < 0.01) and 48 h (*p* < 0.01). Figure 3f shows that the protein expression of those five proteins generally followed the mRNA trends. The results indicated that ZOL would cause the alterations of TMJOA ECM-related gene and protein expression and induce chondrocyte degradation.

### 3.3 Proapoptotic and anti-proliferation effects of ZOL on IL-1β‑induced TMJOA chondrocytes

**Figure 4:**
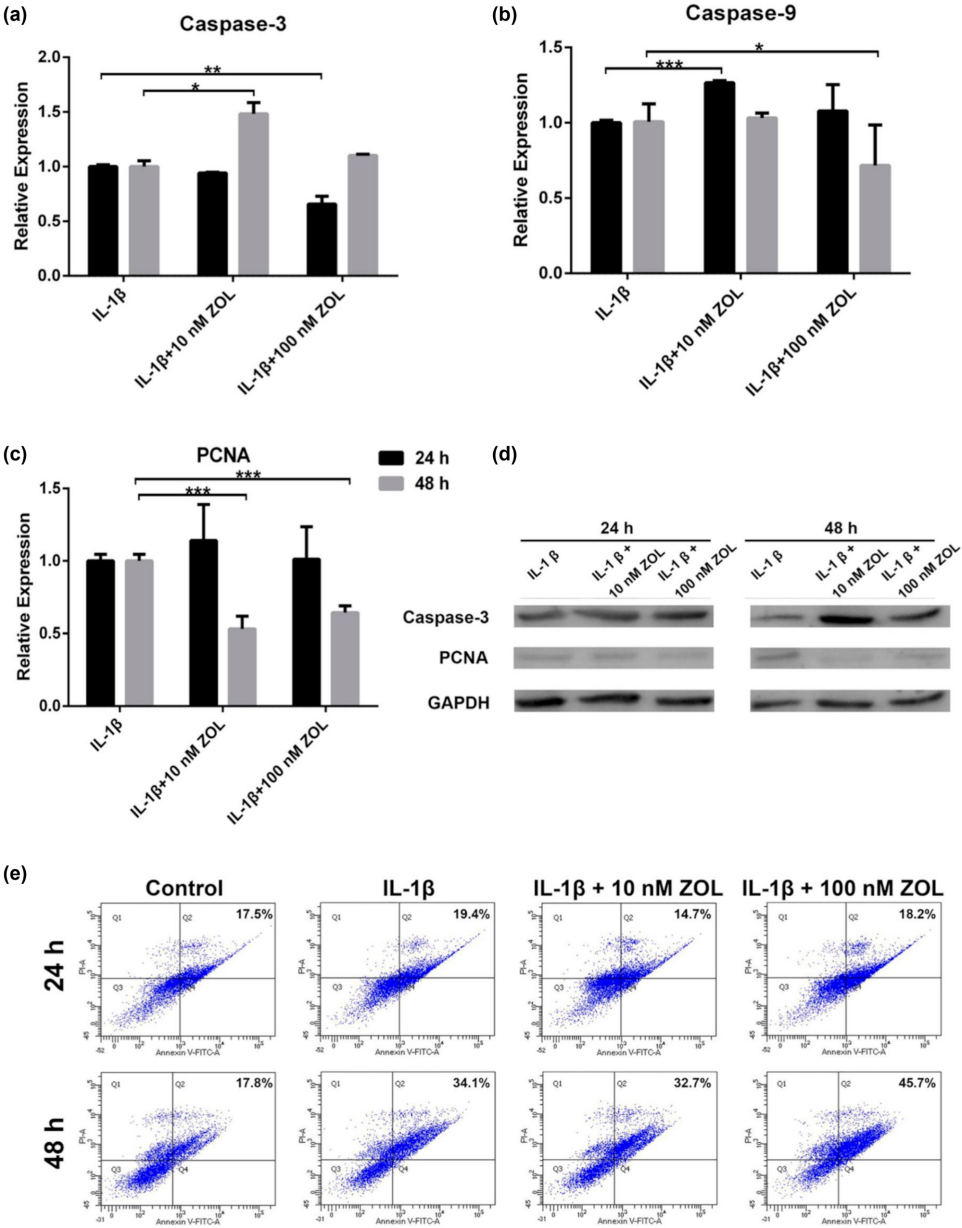
Expression levels of apoptosis- and proliferation-related genes, proteins, and percentage of apoptotic cells after treatment with 10 or 100 nM ZOL in IMCCs. (a) Caspase-3, (b) Caspase-9, and (c) PCNA were analyzed by qRT-PCR. (d) Caspase-3 and PCNA proteins were detected by western blotting. (e) Flow cytometry analysis for the apoptotic cells. All data are presented as mean ± SEM. *p < 0.05, **p < 0.01, ***p < 0.001 between the two groups linked by the line; all other comparisons are not significant.

To assess the effect of ZOL on the apoptosis and proliferation of IL-1β‑induced TMJOA chondrocytes, the mRNA and protein expression levels of caspase-3, caspase-9, and PCNA were determined. It was observed that the mRNA expression levels of caspase-3 and Caspase-9 were upregulated when treated with 10 nM for 24 (*p* < 0.05) and 48 h (*p* < 0.001), respectively (Figure 4a and b). The mRNA expression of PCNA was significantly downregulated when the cells were treated with 10 and 100 nM (both *p* < 0.001) ZOL for 48 h (Figure 4c). Caspase-3 was upregulated, and PCNA was downregulated with ZOL treatment, independently of time and concentration (Figure 4d). Flow cytometry was performed to verify whether ZOL would affect cell apoptosis in TMJOA cells. The results showed no obvious differences after treatment for 24 h no matter which concentration, but after 48 h, the apoptotic rate increased from 34.1 to 45.7% in response to 100 nM ZOL (Figure 4e). It indicates that ZOL could induce TMJOA cell apoptosis in a dose- and time-dependent manner. When combining those results, ZOL could induce TMJOA cell apoptosis by upregulating caspase genes and suppressing the proliferation gene, especially with 100 nM for 48 h.

### 3.4 ZOL affects chondrocyte degradation, involving the Wnt/β-catenin signaling pathway

**Figure 5:**
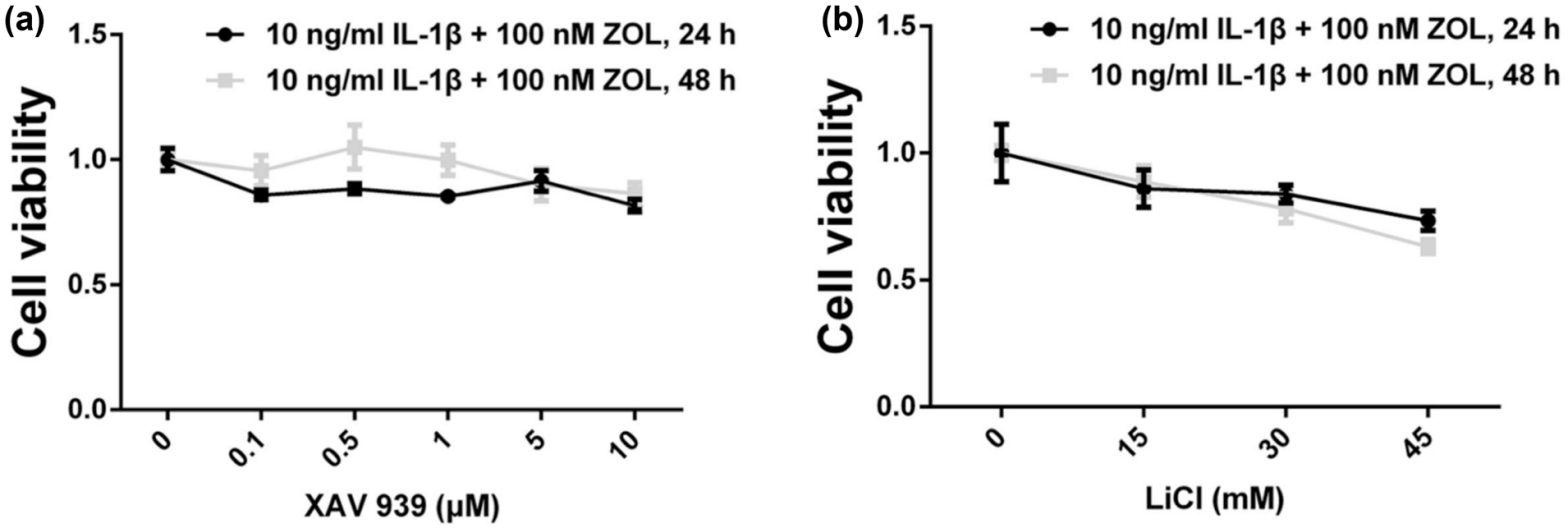
Determination of the concentrations of XAV-939 and LiCl. Cell viability was measured by the MTT assay after XAV-939 (a) or LiCl (b) and 100 nM ZOL treatment of TMJOA cells for 48 h.

We performed the MTT viability assay to select appropriate concentrations of XAV-939 and LiCl. According to the trend in cell viability (Figure 5a and b) and previous studies [30,31], we selected 1 µM XAV-939 and 45 mM LiCl for the subsequent experiments.

**Figure 6:**
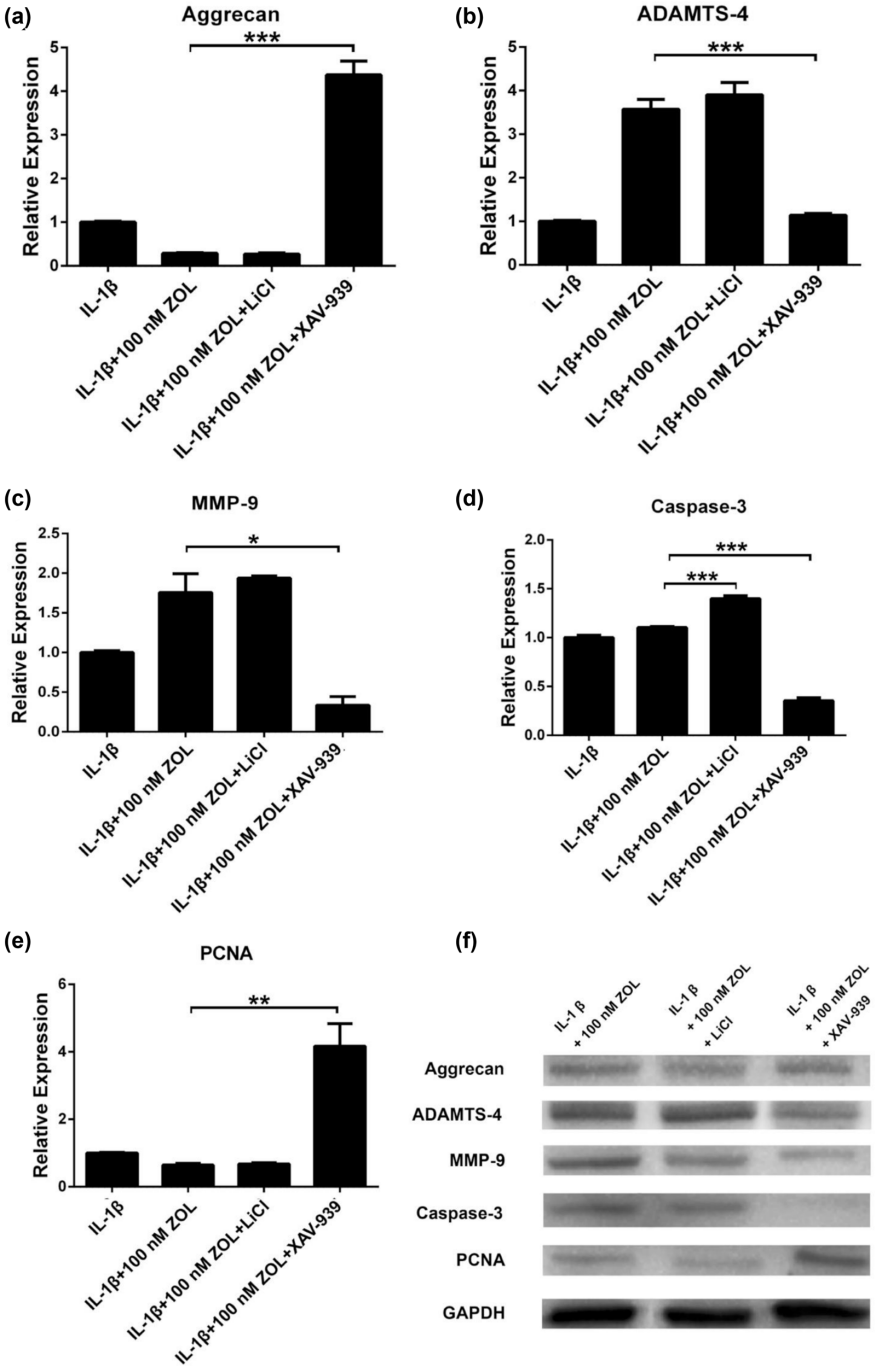
Involvement of Wnt/β-catenin signaling in the effects of ZOL to TMJOA degradation. Relative mRNA expression levels of (a) aggrecan, (b) ADAMTS-4, (c) MMP-9, (d) Caspase-3, and (e) PCNA were analyzed by qRT-PCR. (f) Western blotting analysis of those proteins. All data are presented as mean ± SEM. *p < 0.05, **p < 0.01, ***p < 0.001 between the two groups linked by the line; all other comparisons are not significant.

After treating TMJOA chondrocytes with 100 nM ZOL for 48 h, LiCl was added to activate the Wnt/β-catenin signaling. Aggrecan, ADAMTS-4, and MMP-9 showed no changes compared with ZOL alone. On the other hand, the Wnt/β-catenin signaling inhibitor (XAV-939) upregulated aggrecan (*p* < 0.001) and downregulated ADAMTS-4 (*p* < 0.001) and MMP-9 (*p* < 0.001) (Figure 6a–c). Caspase-3 was upregulated by LiCl and downregulated by XAV-939 (*p* < 0.001) (Figure 6d). PCNA was upregulated with XAV-939 treatment (*p* < 0.01) (Figure 6e). Western blotting was conducted to examine the possible involvement of the Wnt/β-catenin signaling in the ZOL-induced regulation of chondrocytes degradation, and the results were consistent with the mRNA changes (Figure 6f). The results indicate that the effects of ZOL on TMJOA could involve the Wnt/β-catenin signaling pathway.

## 4 Discussion

Few studies focused on the effects of ZOL on chondrocytes, especially on TMJOA chondrocytes, ECM degradation, apoptosis, and related mechanisms. This study examined the role and potential mechanism of ZOL on IL-1β-induced TMJOA chondrocytes. The results suggest that ZOL enhances the IL-1β-induced ECM degradation and cell apoptosis in TMJOA chondrocyte models. The results also suggest that Wnt/β-catenin signaling might be involved in this process, but additional studies are necessary to determine the exact involvement.

This study showed that IL-1β, as an inflammatory activator, increased the production of ECM by chondrocytes, which would be conducive to the development of OA while ZOL decreased ECM production, increased ECM degradation, and induced the apoptosis of chondrocytes, especially at a high concentration and over a long-term treatment in IL-1β-induced TMJOA chondrocyte model. On the other hand, Cinar et al. [20] showed that ZOL had chondroprotective effects in rats, which was also observed in dogs [22]. Nevertheless, direct comparisons among these studies are difficult since the type and activity of the BPs used in those studies are different, the models are different, and the treatment time and doses are different. Therefore, considering those conflicting results, studies are still necessary to determine the effect of ZOL on TMJOA. It is possible that different joints react differently to ZOL because of different conditions like weight-bearing. Studies specifically aiming at TMJOA are necessary.

Studies showed that high-dose ZOL by intravenous injection might be chondroprotective in an OA animal model [21,22]. A recent meta-analysis draws a contradictory conclusion to prior reviews that BPs could not provide symptomatic relief or defer radiographic progression in knee OA [23], as supported by a more recent trial [32]. On other aspects, ZOL could inhibit the growth of hematopoietic cell transplant 116 colorectal cancer cells *in vitro* and *in vivo* and induce apoptosis through the mitochondria pathway [24]. ZOL also has antiproliferative and proapoptotic effects in dental pulp stem cells [25]. Yi et al. [19] showed that ZOL modulated the expression of VEGF-a and could affect the progression of OA. Bagi et al. [33] showed that BPs could not slow the progression of OA in rat models with traumatic OA. A recent study showed that ZOL had protective effects in rabbit OA models [34].

Wnt signaling is a conserved pathway associated with the response to cell differentiation and fate determination during embryogenesis and the late stages of development [35]. Evidence suggests that the activation of the Wnt/β-catenin signaling, the canonical pathway, could be involved in cartilage destruction in arthritis [14,36]. Notably, in this study, when Wnt/β-catenin signaling was activated, the expression levels of apoptosis genes were increased in ZOL-treated TMJOA chondrocytes, and when it was inhibited, ECM-related genes and proteins were increased and apoptosis caused by ZOL was reversed. Those results suggest the involvement of the Wnt/β-catenin signaling in TMJOA and the response of TMJOA chondrocytes to ZOL, but the determination of the exact regulation of Wnt/β-catenin signaling will require additional studies, including gene knockout, overexpression, and silencing.

It is interesting to note that caspase-3 decreased in response to IL-1β + 100 nM ZOL compared with IL-1β + 10 nM ZOL (Figure 4d), but apoptosis increased in response to IL-1β + 100 nM ZOL compared with IL-1β + 10 nM ZOL (Figure 4e) for 48 h. These results indicated that the apoptosis of chondrocytes was not only regulated by caspase-3 but also by other apoptosis factors that were not examined in this study. This study indicated that 48 h and a high concentration of ZOL can promote the apoptosis of chondrocytes, which may be affected by caspase-3, but future studies will have to examine multiple proteins involved in apoptosis.

This study has limitations. It was performed in cells, and the translation of the results to an *in vivo* setting and humans is unknown. In addition, no blank group was set up in the study design. Only a small panel of genes and proteins was investigated, and the exact mechanisms are not elucidated. Examining those pathways will have to be carried out in future studies. Finally, animal studies will have to be performed to verify the results and determine the effects of ZOL on joint development and joint diseases.

In conclusion, this is the first study that investigated the changes in aggrecan, collagen II, matrix metalloproteinase, and chondrocyte apoptosis in IL-1β-treated TMJ chondrocytes after ZOL treatment, and found that ZOL would enhance ECM degradation, promote cell apoptosis, and suppress cell proliferation in rabbit TMJOA cells and that these effects might involve the Wnt/β-catenin signaling, but additional studies are necessary for confirmation of the exact involvement of Wnt/β-catenin signaling in chondrocytes after ZOL treatment.

## Abbreviations

TMJ: temporomandibular joint
TMJOA: temporomandibular joint osteoarthritis
ECM: extracellular matrix
IMCCs: immortalized mandibular condylar chondrocytes
PCNA: proliferating cell nuclear antigen
OA: osteoarthritis
TMD: temporomandibular disorders
IL: interleukin
MMPs: matrix metalloproteases
ADAMTS: a disintegrin and metalloproteinase with thrombospondin motifs
BPs: bisphosphonates
BRONJ: BP-related osteonecrosis of the jaw
IMCC: immortalized mandibular condylar chondrocyte
qRT-PCR: reverse transcription-quantitative polymerase chain reaction
ZOL: zoledronate

## References

[1] Lim WH, Toothman J, Miller JH, Tallents RH, Brouxhon SM, Olschowka ME, et al. IL-1beta inhibits TGFbeta in the temporomandibular joint. J Dent Res. 2009;88(6):557–62. 10.1177/0022034509336823. PMC3317986 19587162

[2] Tanaka E, Detamore MS, Mercuri LG. Degenerative disorders of the temporomandibular joint: etiology, diagnosis, and treatment. J Dent Res. 2008;87(4):296–307. 10.1177/154405910808700406. 18362309

[3] Zarb GA, Carlsson GE. Temporomandibular disorders: osteoarthritis. J Orofac Pain. 1999;13(4):295–306. 10823044

[4] Vernal R, Velasquez E, Gamonal J, Garcia-Sanz JA, Silva A, Sanz M. Expression of proinflammatory cytokines in osteoarthritis of the temporomandibular joint. Arch Oral Biol. 2008;53(10):910–5. 10.1016/j.archoralbio.2008.04.004. 18508030

[5] Cevidanes LH, Walker D, Schilling J, Sugai J, Giannobile W, Paniagua B, et al. 3D osteoarthritic changes in TMJ condylar morphology correlates with specific systemic and local biomarkers of disease. Osteoarthritis Cartilage. 2014;22(10):1657–67. 10.1016/j.joca.2014.06.014. PMC4185299 25278075

[6] Li W, Wu M, Jiang S, Ding W, Luo Q, Shi J. Expression of ADAMTs-5 and TIMP-3 in the condylar cartilage of rats induced by experimentally created osteoarthritis. Arch Oral Biol. 2014;59(5):524–9. 10.1016/j.archoralbio.2014.02.016. 24632095

[7] Ge XP, Gan YH, Zhang CG, Zhou CY, Ma KT, Meng JH, et al. Requirement of the NF-kappaB pathway for induction of Wnt-5A by interleukin-1beta in condylar chondrocytes of the temporomandibular joint: functional crosstalk between the Wnt-5A and NF-kappaB signaling pathways. Osteoarthritis Cartilage. 2011;19(1):111–7. 10.1016/j.joca.2010.10.016. 21035559

[8] Aida Y, Maeno M, Suzuki N, Shiratsuchi H, Motohashi M, Matsumura H. The effect of IL-1beta on the expression of matrix metalloproteinases and tissue inhibitors of matrix metalloproteinases in human chondrocytes. Life Sci. 2005;77(25):3210–21. 10.1016/j.lfs.2005.05.052. 15979654

[9] Zhou PH, Liu SQ, Peng H. The effect of hyaluronic acid on IL-1beta-induced chondrocyte apoptosis in a rat model of osteoarthritis. J Orthop Res. 2008;26(12):1643–8. 10.1002/jor.20683. 18524010

[10] Doege KJ, Sasaki M, Kimura T, Yamada Y. Complete coding sequence and deduced primary structure of the human cartilage large aggregating proteoglycan, aggrecan. Human-specific repeats, and additional alternatively spliced forms. J Biol Chem. 1991;266(2):894–902. 1985970

[11] Luo Y, Sinkeviciute D, He Y, Karsdal M, Henrotin Y, Mobasheri A, et al. The minor collagens in articular cartilage. Protein Cell. 2017;8(8):560–72. 10.1007/s13238-017-0377-7. PMC5546929 28213717

[12] Zhu M, Tang D, Wu Q, Hao S, Chen M, Xie C, et al. Activation of beta-catenin signaling in articular chondrocytes leads to osteoarthritis-like phenotype in adult beta-catenin conditional activation mice. J Bone Miner Res. 2009;24(1):12–21. 10.1359/jbmr.080901. PMC2640321 18767925

[13] Landman EB, Miclea RL, van Blitterswijk CA, Karperien M. Small molecule inhibitors of WNT/beta-catenin signaling block IL-1beta- and TNFalpha-induced cartilage degradation. Arthritis Res Ther. 2013;15(4):R93. 10.1186/ar4273. PMC3978727 23965253

[14] Corr M. Wnt-beta-catenin signaling in the pathogenesis of osteoarthritis. Nat Clin Pract Rheumatol. 2008;4(10):550–6. 10.1038/ncprheum0904. 18820702

[15] Zhou Y, Wang T, Hamilton JL, Chen D. Wnt/beta-catenin signaling in osteoarthritis and in other forms of arthritis. Curr Rheumatol Rep. 2017;19(9):53. 10.1007/s11926-017-0679-z. PMC5672801 28752488

[16] Wang Y, Fan X, Xing L, Tian F. Wnt signaling: a promising target for osteoarthritis therapy. Cell Commun Signal. 2019;17(1):97. 10.1186/s12964-019-0411-x. PMC6697957 31420042

[17] Green JR. Preclinical pharmacology of zoledronic acid. Semin Oncol. 2002;29(6 Suppl 21):3–11. 10.1053/sonc.2002.37421. 12584689

[18] Van Offel JF, Schuerwegh AJ, Bridts CH, Stevens WJ, De Clerck LS. Effect of bisphosphonates on viability, proliferation, and dexamethasone-induced apoptosis of articular chondrocytes. Ann Rheum Dis. 2002;61(10):925–8. 10.1136/ard.61.10.925. PMC1753899 12228165

[19] Yi JW, Lee WS, Kim SB, Heo YM, Chae DS. Effect of zoledronate on the expression of vascular endothelial growth factor-a by articular chondrocytes and synovial cells: an in vitro study. J Bone Metab. 2014;21(4):249–55. 10.11005/jbm.2014.21.4.249. PMC4255045 25489573

[20] Cinar BM, Ozkoc G, Bolat F, Karaeminogullari O, Sezgin N, Tandogan RN. Intra-articular zoledronic acid in a rat osteoarthritis model: significant reduced synovitis may indicate chondroprotective effect. Knee Surg Sports Traumatol Arthrosc. 2015;23(5):1410–8. 10.1007/s00167-014-2955-z. 24664185

[21] Lampropoulou-Adamidou K, Dontas I, Stathopoulos IP, Khaldi L, Lelovas P, Vlamis J, et al. Chondroprotective effect of high-dose zoledronic acid: An experimental study in a rabbit model of osteoarthritis. J Orthop Res. 2014;32(12):1646–51. 10.1002/jor.22712. 25125266

[22] Dearmin MG, Trumble TN, Garcia A, Chambers JN, Budsberg SC. Chondroprotective effects of zoledronic acid on articular cartilage in dogs with experimentally induced osteoarthritis. Am J Vet Res. 2014;75(4):329–37. 10.2460/ajvr.75.4.329. 24669915

[23] Vaysbrot EE, Osani MC, Musetti MC, McAlindon TE, Bannuru RR. Are bisphosphonates efficacious in knee osteoarthritis? A meta-analysis of randomized controlled trials. Osteoarthritis Cartilage. 2018;26(2):154–64. 10.1016/j.joca.2017.11.013. 29222056

[24] Gao X, Jiang B, Zou S, Zhang T, Qi X, Jin L, et al. Zoledronate can promote apoptosis and inhibit the proliferation of colorectal cancer cells. Tumour Biol. 2015;36(7):5315–22. 10.1007/s13277-015-3192-x. 25682285

[25] Pourgonabadi S, Mousavi SH, Tayarani-Najaran Z, Ghorbani A. Effect of zoledronate, a third-generation bisphosphonate, on proliferation and apoptosis of human dental pulp stem cells. Can J Physiol Pharmacol. 2018;96(2):137–44. 10.1139/cjpp-2016-0348. 28772088

[26] Hammarfjord O, Stassen LF. Bisphosphonate therapy and ankylosis of the temporomandibular joint: is there a relationship? A case report. Oral Surg Oral Med Oral Pathol Oral Radiol. 2014;118(3):e68–70. 10.1016/j.oooo.2014.02.011. 24906945

[27] Enomoto A, Uchihashi T, Izumoto T, Nakahara H, Hamada S. Suppurative arthritis of the temporomandibular joint associated with bisphosphonate: a case report. J Oral Maxillofac Surg. 2012;70(6):1376–9. 10.1016/j.joms.2011.06.215. 21855198

[28] Ozkan G, Demetoglu U, Simsek HO, Semetoglu GA. Management of temporomandibular joint dislocation in a bisphosphonate-medicated patient. Austin J Radiol. 2016;3(3):1055.

[29] Wang SC. PCNA: a silent housekeeper or a potential therapeutic target? Trends Pharmacol Sci. 2014;35(4):178–86. 10.1016/j.tips.2014.02.004. 24655521

[30] Fu HD, Wang HR, Li DH. BMP-7 accelerates the differentiation of rabbit mesenchymal stem cells into cartilage through the Wnt/beta-catenin pathway. Exp Ther Med. 2017;14(6):5424–8. 10.3892/etm.2017.5210. PMC5740575 29285071

[31] Xia MY, Zhao XY, Huang QL, Sun HY, Sun C, Yuan J, et al. Activation of Wnt/beta-catenin signaling by lithium chloride attenuates d-galactose-induced neurodegeneration in the auditory cortex of a rat model of aging. FEBS Open Bio. 2017;7(6):759–76. 10.1002/2211-5463.12220. PMC5458451 28593132

[32] Cai G, Aitken D, Laslett LL, Pelletier JP, Martel-Pelletier J, Hill C, et al. Effect of intravenous zoledronic acid on tibiofemoral cartilage volume among patients with knee osteoarthritis with bone marrow lesions: a randomized clinical trial. JAMA. 2020;323(15):1456–66. 10.1001/jama.2020.2938. PMC7175085 32315057

[33] Bagi CM, Berryman E, Zakur DE, Wilkie D, Andresen CJ. Effect of antiresorptive and anabolic bone therapy on development of osteoarthritis in a posttraumatic rat model of OA. Arthritis Res Ther. 2015;17:315. 10.1186/s13075-015-0829-5. PMC4635572 26542671

[34] She G, Zhou Z, Zha Z, Wang F, Pan X. Protective effect of zoledronic acid on articular cartilage and subchondral bone of rabbits with experimental knee osteoarthritis. Exp Ther Med. 2017;14(5):4901–9. 10.3892/etm.2017.5135. PMC5704325 29201194

[35] Sassi N, Laadhar L, Allouche M, Achek A, Kallel-Sellami M, Makni S, et al. WNT signaling and chondrocytes: from cell fate determination to osteoarthritis physiopathology. J Recept Signal Transduct Res. 2014;34(2):73–80. 10.3109/10799893.2013.863919. 24303940

[36] Luyten FP, Tylzanowski P, Lories RJ. Wnt signaling and osteoarthritis. Bone. 2009;44(4):522–7. 10.1016/j.bone.2008.12.006. 19136083

